# Polygenic risk of refractory celiac disease type II and its association with autoimmune diseases: a phenome-wide association study in the UK Biobank

**DOI:** 10.1097/MEG.0000000000003036

**Published:** 2025-07-14

**Authors:** Lampriani Tsali, Konstantinos Tsilidis, Konstantinos Katsanos, Evangelia Ntzani, Maria Manou, Christopher Papandreou, Georgios Markozannes, Christos V. Chalitsios

**Affiliations:** a Department of Hygiene and Epidemiology, University of Ioannina School of Medicine, Ioannina, Greece; b Department of Epidemiology and Biostatistics, School of Public Health, Imperial College London, London, UK; c Division of Gastroenterology, Department of Internal Medicine, Faculty of Medicine, School of Health Sciences, University of Ioannina, Ioannina, Greece; d Department of Health Services, Policy and Practice, School of Public Health, Center for Evidence-Based Medicine, Brown University, Providence, Rhode Island, USA; e Institute of Health Pere Virgili (IISPV), Reus, Spain; f Department of Nutrition and Dietetics Sciences, School of Health Sciences Hellenic Mediterranean University (HMU), Siteia, Greece; g Nuffield Department of Clinical Neurosciences, University of Oxford, Oxford, UK

**Keywords:** ankylosing spondylitis, autoimmune disease, phenome-wide, polygenic risk score, refractory celiac disease, UK Biobank

## Abstract

**Objective:**

Refractory celiac disease-type II (RCDII) is the more severe and adverse form of celiac disease; however, its association with other autoimmune diseases remains unclear. We conducted a phenome-wide association study (PheWAS) to examine the association between the polygenic risk score (PRS) for RCDII and autoimmune diseases.

**Methods:**

To construct the PRS-RCDII, we extracted summary statistics for three non-human leukocyte antigen genetic variants, which were independently associated with RCDII (*r*^2^ < 0.001; *P* < 5 × 10^−5^) in a genome-wide association study. We then conducted a PRS-PheWAS in the UK Biobank to investigate the associations of PRS-RCDII with 27 autoimmune diseases, adjusting for age, sex, genetic batch, and genetic ancestry. False discovery rate (FDR < 0.05) correction was applied to account for multiple comparisons.

**Results:**

Our study population comprised 373 022 UK Biobank participants (mean age: 57.2 years), of whom 202 865 (54.4%) were females. We constructed the PRS-RCDII, using three genetic variants, namely rs2041570 on chromosome 7p14.3 (*FAM188B*), rs7324708 on chromosome 13q22.1 (*KLF12*), and rs205047 on chromosome 17p12 (*SHISA6*). In the PRS-PheWAS, two phenotypes were initially associated with RCDII at a nominal *P* value threshold, ankylosing spondylitis and systemic sclerosis; however, after adjusting for multiple comparisons, only the association with ankylosing spondylitis remained statistically significant (odds ratio_per 1 SD increase_ = 1.13; 95% confidence interval: 1.04–1.22; *P*_FDR_ = 0.023). Sex-stratified and single-nucleotide polymorphism (SNP)-by-SNP analyses revealed no significant heterogeneity.

**Conclusion:**

Our study identified an association between the genetic risk score for RCDII and ankylosing spondylitis, but not with other autoimmune diseases. This finding may have clinical importance for people with RCDII, although replication in future studies is needed.

## Introduction

Celiac disease (CeD) is a chronic, immune-mediated medical condition that affects approximately 1% of the general population [1]. It can be effectively controlled and treated with a long-life gluten-free diet (GFD) and has been associated with nongenetic [2] and genetic risk factors. Genetic heritability includes a combination of human leukocyte antigen (HLA) and non-HLA genetic factors [3]. CeD co-occurs with other autoimmune diseases in approximately 20% of adults and expands with increasing age at diagnosis [4,5]. The association of CeD with autoimmune disorders has been extensively studied in the literature for many autoimmune entities [6–16]. A recently published Mendelian randomization-phenome-wide association study (MR-PheWAS) suggested a potential causal association of CeD with type 1 diabetes, Graves’ disease, Sjögren syndrome, chronic hepatitis, systemic and cutaneous lupus erythematosus, and sarcoidosis [17].

However, little is known about the more aggressive and potentially lethal form of CeD, refractory celiac disease (RCD), and its genetic association with autoimmune disorders. RCD accounts for approximately 1–5% of people with CeD who do not respond to GFD and progress to RCD type I or type II (RCDI or RCDII) [18]. The latter is a more serious condition as it is associated with a poor prognosis, a high mortality rate, and a 5-year survival rate of less than 50% [19]. Specifically, people with RCDII show persistence of malabsorption after 12 months on a strict GFD and intestinal damage histologically, and the majority of them progress to more severe disease through the development of aggressive enteropathy-associated T-cell lymphoma [19].

Between 44 and 60% of people with RCDII are HLA-DQ2 homozygous [20], and rs7259292 in *MYO9B* has been associated with an increased risk of developing the disease [21]. Studies on the pathogenesis of RCDII have highlighted the role of cytokines such as interleukin (IL)-15, IL-21, IL-7, as well as mutations in the Janus kinase/signal transducer and activator of transcription pathway [22]. In addition, a genome-wide association study (GWAS) on RCDII showed that genetic susceptibility and variants associated with CeD might differ from those associated with RCDII [23]. Therefore, observing the current gap in understanding the genetic underpinnings of RCDII and understanding the need for further research in the area, we aimed to investigate whether a higher genetic risk of RCDII is associated with autoimmune diseases in the UK Biobank cohort.

## Methods

### Polygenic risk score

**Fig. 1.**
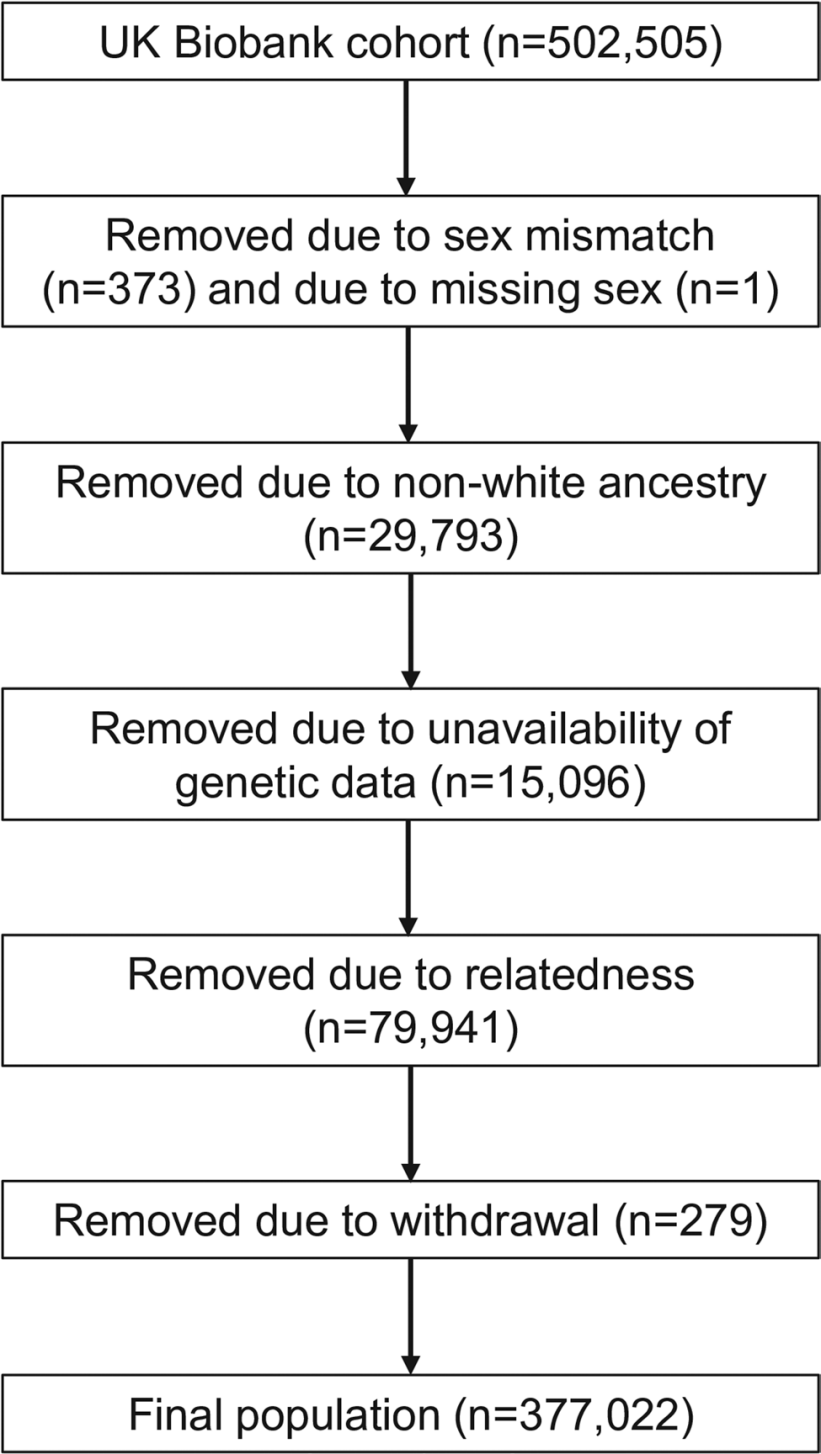
Participant selection flowchart.

We included single-nucleotide polymorphisms (SNPs) from a meta-analysis of GWAS of European participants (*n*_cases_ = 94, *n*_controls_ = 2131) of non-HLA genetic variants [23] associated with RCDII at *P* less than 5 × 10^–5^ (consistent with the original GWAS reporting and given the limited sample size and rarity of RCDII) to create a standardized weighted PRS. We calculated a weighted polygenic risk score (PRS) for RCDII for each participant in the final analytical sample of the UK Biobank, following the application of exclusion criteria (Fig. 1), using PLINK (version 1.9). The score was derived by summing the number of RCDII-increasing alleles multiplied by their corresponding beta estimates. The PRS was then standardized by subtracting the mean (0.19) and dividing by the SD (SD = 0.13) of its distribution to facilitate interpretation.

### Phenome-wide association study

#### Data sources and study population

In the current analysis, we used the UK Biobank [24], a large prospective cohort study that enrolled over 500 000 participants residents in the UK aged 37–73 years at the time of recruitment. Baseline and subsequent self-reported measurements of various anthropometric, lifestyle, and dietary variables were recorded. The participants provided blood samples for genotyping and biochemical analyses. Each participant’s data are linked to electronic health data, including cancer registry, death registry, and the hospital episode statistics (HES) [25], a national medical data warehouse encompassing patients’ clinical appointments and hospital admissions within the UK.

We constrained our analyses to white-unrelated individuals of European ancestry with available genetic data (Fig. 1). To control for sample relatedness in our analyses, we randomly excluded one participant from each pair of relatives based on a kinship coefficient greater than 0.0884 as provided by the UK Biobank.

#### Outcome definition

Disease outcomes were captured by inpatient HES. Disease registry data were used to ascertain the respective disease outcomes. These datasets contain conditions coded according to the International Classification of Diseases (ICD) coding system, especially the ninth (ICD-9) or 10^th^ (ICD-10) revisions. We formatted ICD-9 and ICD-10 to the phecode grouping system [26,27], as implemented in the PheWAS R package [28]. Phecodes correspond to groups of disease codes associated with diseases in clinical practice and genomic studies. For each phecode, the respective case-control groups were created. Cases were defined as participants presenting the phecode of interest, while controls were defined by the absence of the tested or related phecodes. Converting ICD-9 or ICD-10 codes to phecodes resulted in 27 distinct phecodes. The selected phecodes correspond to the most frequent autoimmune diseases associated in the literature with CeD or RCD (Table S1, Supplemental digital content 1, https://links.lww.com/EJGH/B193).

### Statistical analysis

We used the ‘PheWAS’ package in R to perform a logistic regression of each autoimmune disease phecode, adjusting for age (years), sex (male and female), genetic batch, and the first 15 principal components of genetic ancestry. Sex-stratified analyses were also conducted. In addition, we conducted a SNP-by-SNP analysis to identify potential heterogeneity between PRS-RCDII and the statistically significant outcomes. Because many phecodes are not independent, we also applied the false discovery rate (FDR < 0.05) correction to account for multiple testing following the Benjamini–Hochberg procedure [29].

### Ethical approval

The UK Biobank study was approved by the North West Multi-Centre Research Ethics Committee on 17 June 2011 under the reference 11/NW/0382. All the participants in the UK Biobank study provided written informed consent. Our analysis was undertaken under the application number 79696.

## Results

Our study population comprised 373 022 UK Biobank participants (mean age: 57.2 years), of whom 202 865 (54.4%) were females (Table 1). We examined the association of the PRS-RCDII, derived from three non-HLA genetic variants, namely rs2041570 on chromosome 7p14.3 (*FAM188B*), rs7324708 on chromosome 13q22.1 (*KLF12*), and rs205047 on chromosome 17p12 (*SHISA6*), with 27 unique phecodes related to autoimmune diseases.

**Table 1.** Baseline characteristics of the study population in the UK Biobank

	Total (*N* = 373 022)
Sex, females	202 865 (54.4)
Age, mean (SD), (years)	57.2 (7.9)
Townsend deprivation index, median (IQR)	−2.2 (−3.7 to 0.4)
Physical activity, median (IQR), (h/week)	1784 (813–3573)
BMI, mean (SD), (kg/m^2^)	27.3 (4.7)
Smoking status	
Current	37 126 (10)
Former	133 560 (35.8)
Never	202 336 (54.2)

All figures are expressed as absolute numbers (percentages, %) unless otherwise specified.

IQR, interquartile range.

**Fig. 2.**
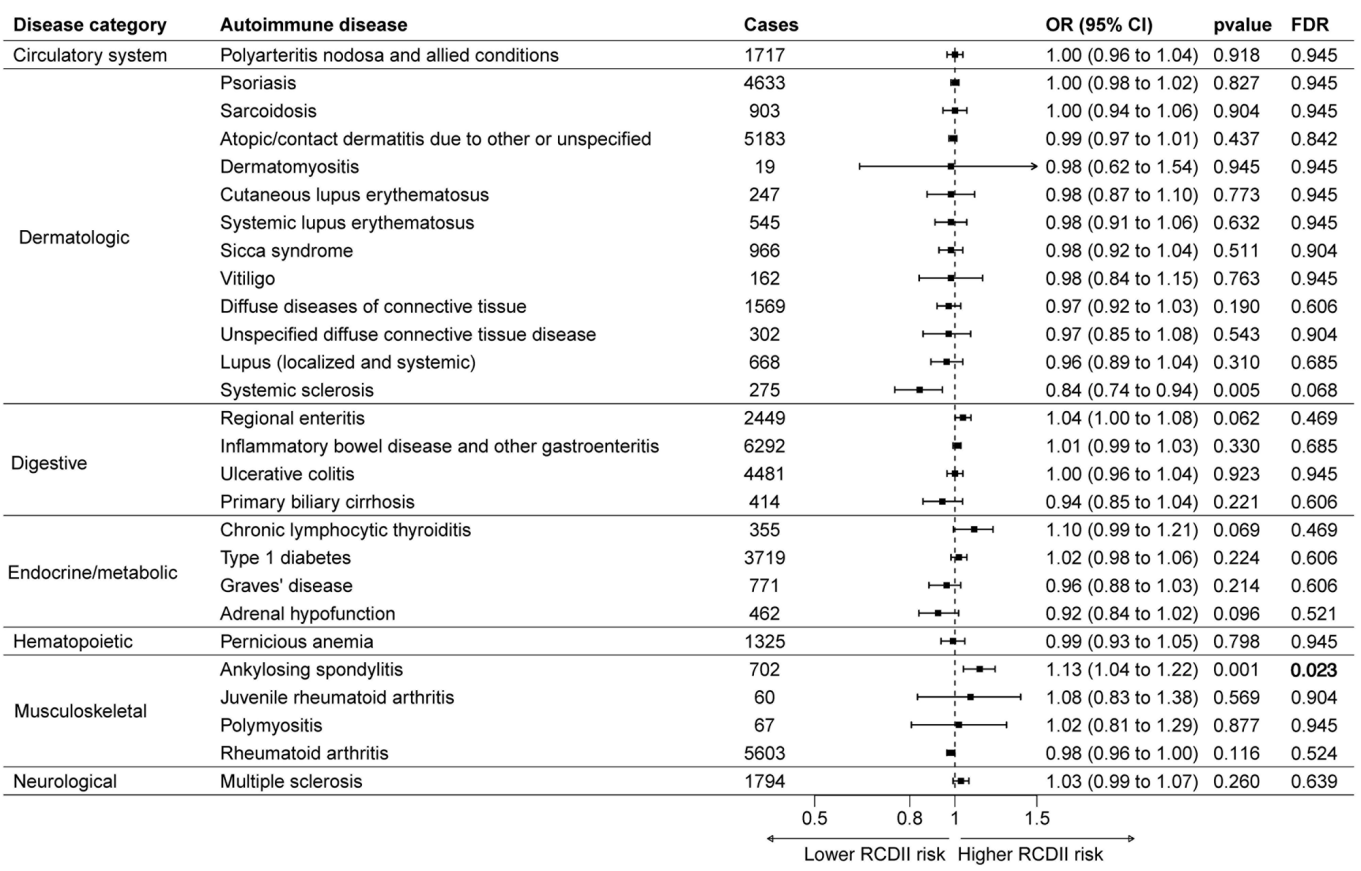
Associations between polygenic risk score of refractory celiac disease-type II and 27 autoimmune diseases in the UK Biobank. CI, confidence interval; FDR, false discovery rate; OR, odds ratio; RCDII, refractory celiac disease-type II.

In the PRS-PheWAS, two phenotypes (ankylosing spondylitis and systemic sclerosis) were associated with RCDII at the nominal *P* value threshold; however, after adjusting for multiple comparisons (FDR correction), only the association with ankylosing spondylitis remained statistically significant [odds ratio (OR)_per 1SD increase_ = 1.13; 95% confidence interval (CI): 1.04–1.22; *P*_FDR_ = 0.023) (Fig. 2, Table S1, Supplemental digital content 1, https://links.lww.com/EJGH/B193). Sex-stratified analyses revealed no significant differences in the association between the PRS and RCDII in males or females (Tables S2 and S3, Supplemental digital content 1, https://links.lww.com/EJGH/B193). Finally, Table S4, Supplemental digital content 1, https://links.lww.com/EJGH/B193 summarizes association results for individual SNPs with ankylosing spondylitis and systemic sclerosis, showing no evidence of heterogeneity across SNPs. No other autoimmune diseases were associated with the PRS-RCDII.

## Discussion

In this study, we employed a data-driven approach to assess whether a higher genetic predisposition to RCDII is associated with autoimmune diseases by constructing the PRS-RCDII and then conducting a PRS-PheWAS in the UK Biobank cohort. We detected a positive association between a higher genetic risk of RCDII and ankylosing spondylitis, but not with other autoimmune diseases. Sex-stratified analyses revealed no significant differences.

The observed association between genetic risk for RCDII and ankylosing spondylitis carries potential clinical implications. To date, no prior studies have examined this association, leaving our findings without direct comparison. The most common form of CeD is associated with various extraintestinal manifestations, including connective tissue diseases, which occur in up to 9% of patients. Arthritis is also recognized as an extraintestinal manifestation of CeD [30]. Nonetheless, nothing is known about the specific association of RCDII with ankylosing spondylitis; thus, this field is unexplored to date.

Ankylosing spondylitis is a chronic, lifelong inflammatory disease that, while not directly life-threatening, can significantly impact overall survival and quality of life [31], particularly when co-occurring with RCDII. In this context, patients not only face the challenges of RCD, characterized by persistent gastrointestinal symptoms unresponsive to a GFD and severe RCDII-related complications such as lymphoma, gastrointestinal malabsorption, and secondary malignancies [32], but must also contend with the debilitating morbidities associated with ankylosing spondylitis. These include progressive ankylosis, severe chronic pain, and stiffness, primarily affecting the spine and sacroiliac joints [31].

The function of the three non-HLA SNPs that were used in the PRS for RCDII can provide interesting suggestions for potential biological mechanisms linking RCDII with ankylosing spondylitis. In addition to its known association with progression to RCDII, potentially through its regulation of *FAM188B* expression in immune-related tissues, rs2041570 has also been associated with increased expression of *C20orf114 (BPIFB1*) and alterations in intestinal epithelial innate immune genes and antimicrobial defensins [23]. These defensins, secreted by Paneth cells, contribute to the maintenance of the gastrointestinal barrier. rs7324708, located within *KLF12*, has been associated with variations in lymphocyte count, suggesting a role in immune system regulation. Meanwhile, rs205047, within *SHISA6*, has been implicated in neuronal and immune-related pathways. Notably, alterations in immune responses, intestinal permeability, dysbiosis, and cytokine production because of chronic inflammation have all been proposed as underlying mechanisms in the pathogenesis of ankylosing spondylitis [31] and may represent potential biological links between the two diseases.

RCDII is a resistant disease with a high mortality rate and poor quality of life. It has been observed that long-lasting CeD with delayed diagnosis is characteristic of RCDII [18]. Because ankylosing spondylitis morbidity and sequels may further jeopardize the already poor quality of life of these patients, gastroenterologists and rheumatologists should be aware of this potential connection, either to avoid the progression of CeD to RCDII or to succeed in a prompt diagnosis and management of ankylosing spondylitis disease and symptoms; however, further research is needed to establish the strength and clinical relevance of this association before screening can be formally recommended.

The current study found no associations with the rest of the autoimmune diseases. Most of them have been associated with the more common form of CeD in observational studies and meta-analyses. Specifically, a high prevalence of the more common form of CeD has been found in cohorts of patients with autoimmune diseases such as Hashimoto’s thyroiditis, psoriasis, type 1 diabetes, and Sjögren syndrome [6,14,33,34]. The etiology for the co-occurrence involves the shared HLA genotypes, untreated CeD characterized by altered intestinal permeability and preservation of inflammation, which leads to immune dysregulation by producing proinflammatory cytokines and autoantigens and triggers the nascency of other autoimmune diseases [35]. Although it may seem surprising that no associations were observed with other autoimmune diseases commonly linked to CeD, this finding may point to distinct pathophysiological mechanisms underlying RCDII. The absence of associations likely reflects the use of genetic instruments that are specific to RCDII, rather than markers of general genetic susceptibility to CeD. This is consistent with the different clinical and molecular features of RCDII compared with conventional CeD.

The PRS for RCDII was derived from a GWAS that investigated the non-HLA variations specifically associated with RCDII progression, and found three SNPs associated with this disease, which we use in our work. This enabled the identification of non-HLA genetic variants associated specifically with RCDII, rather than classical CeD, allowing the resulting PRS to capture distinct immunogenetic features of RCDII.

This is the first study examining the association between genetic risk for RCDII and autoimmune diseases. A strength of the study is the investigation of the associations of RCDII with a wide range of autoimmune diseases using a data-driven approach in a large cohort; however, several limitations should be acknowledged in the interpretation of our findings. First, the sample of the RCDII GWAS was small and therefore unable to capture the complete genetic makeup of RCDII; however, three non-HLA SNPs with robust associations were identified, and these SNPs were specific to RCDII, as they were not associated with genetic susceptibility to CeD. In addition, the data in our study pertain to a European population, limiting the generalizability of the findings to other ethnic groups. Our analysis was restricted to individuals of European ancestry, reflecting the populations included in the existing GWAS. While this is not a limitation of our study design per se, it does highlight a broader challenge in the field – the lack of genetic data from diverse populations. The generalizability of our findings in RCDII is inherently constrained until more inclusive genomic studies are undertaken. Furthermore, we were not able to assess potential bidirectional associations between the PRS-ankylosing spondylitis and RCDII, as full summary statistics from the RCDII GWAS were not available. Future research should aim to expand the genetic understanding of RCDII through larger discovery cohorts and investigate whether these genetic associations translate to clinically observable comorbidity patterns. In addition, studies using clinically well-characterised cohorts are warranted to assess the direct association between RCDII and autoimmune diseases.

### Conclusion

Higher genetic risk score for RCDII was positively associated with ankylosing spondylitis, but not with other autoimmune diseases. Given the extreme frailty and poor prognosis of people with RCDII, this association may have clinical significance; however, it requires confirmation in future studies.

## Acknowledgements

This study was conducted under the UK Biobank application number 79696. Access to the data used in this study is subject to approval by the UK Biobank and requires an application process. The data are not publicly available but can be accessed by bona fide researchers through the UK Biobank Access Management System (www.ukbiobank.ac.uk).To construct the polygenic risk score, we used data from a published genome-wide association study (GWAS), which can be accessed in the corresponding publication (PMID: 29787419).

### Conflicts of interest

There are no conflicts of interest.

## Supplementary Material


