# Supplementary material for: Polygenic risk of refractory celiac disease type II and its association with autoimmune diseases: a phenome-wide association study in the UK Biobank
Source: Eur J Gastroenterol Hepatol. 2025 Jul 14;37(12):1344–9. doi: 10.1097/MEG.0000000000003036 (PMC12577659; doi:10.1097/MEG.0000000000003036)
Supplement: Supplementary file 1 [file ejgh-37-1344-s001.pdf]

Table S1. Associations between polygenic risk score of refractory celiac disease-type II and the autoimmune disease phenotypes using the PRS-PheWAS.

Phenotype	Description	Group	OR	95%LCI	95%UCI	P value	FDR	n_cases	n_controls
715.2	Ankylosing spondylitis	musculoskeletal	1.13	1.04	1.22	8.43E-04	**2.28E-02**	702	358978
245.21	Chronic lymphocytic thyroiditis	endocrine/metabolic	1.10	0.99	1.21	6.94E-02	4.69E-01	355	343421
714.2	Juvenile rheumatoid arthritis	musculoskeletal	1.08	0.83	1.38	5.69E-01	9.04E-01	60	358978
555.1	Regional enteritis	Digestive	1.04	1.00	1.08	6.19E-02	4.69E-01	2449	290823
335	Multiple sclerosis	Neurological	1.03	0.99	1.07	2.60E-01	6.39E-01	1794	350511
250.1	Type 1 diabetes	endocrine/metabolic	1.02	0.98	1.06	2.24E-01	6.06E-01	3719	338628
709.4	Polymyositis	Dermatologic	1.02	0.81	1.29	8.77E-01	9.45E-01	67	354765
555	Inflammatory bowel disease and other gastroenteritis and colitis	Digestive	1.01	0.99	1.03	3.30E-01	6.85E-01	6292	290823
696.4	Psoriasis	Dermatologic	1.00	0.98	1.02	8.27E-01	9.45E-01	4633	350520
555.2	Ulcerative colitis	Digestive	1.00	0.96	1.04	9.23E-01	9.45E-01	4481	290823
446	Polyarteritis nodosa and allied conditions	circulatory system	1.00	0.96	1.04	9.18E-01	9.45E-01	1717	356524
697	Sarcoidosis	Dermatologic	1.00	0.94	1.06	9.04E-01	9.45E-01	903	356011
281.11	Pernicious anemia	Hematopoietic	0.99	0.93	1.05	7.98E-01	9.45E-01	1325	335559
939	Atopic/contact dermatitis due to other or unspecified	Dermatologic	0.99	0.97	1.01	4.37E-01	8.42E-01	5183	365119
709.5	Dermatomyositis	Dermatologic	0.98	0.62	1.54	9.45E-01	9.45E-01	19	354765
695.41	Cutaneous lupus erythematosus	Dermatologic	0.98	0.87	1.10	7.73E-01	9.45E-01	247	354330
695.42	Systemic lupus erythematosus	Dermatologic	0.98	0.91	1.06	6.32E-01	9.45E-01	545	354330
714.1	Rheumatoid arthritis	musculoskeletal	0.98	0.96	1.00	1.16E-01	5.24E-01	5603	358978
709.2	Sicca syndrome	Dermatologic	0.98	0.92	1.04	5.11E-01	9.04E-01	966	354765
694.1	Vitiligo	Dermatologic	0.98	0.84	1.15	7.63E-01	9.45E-01	162	356011
709	Diffuse diseases of connective tissue	Dermatologic	0.97	0.92	1.03	1.90E-01	6.06E-01	1569	354765
709.7	Unspecified diffuse connective tissue disease	Dermatologic	0.97	0.85	1.08	5.43E-01	9.04E-01	302	354765
695.4	Lupus (localized and systemic)	Dermatologic	0.96	0.89	1.04	3.10E-01	6.85E-01	668	354330
242.1	Graves' disease	endocrine/metabolic	0.96	0.88	1.03	2.14E-01	6.06E-01	771	343421
571.6	Primary biliary cirrhosis	Digestive	0.94	0.85	1.04	2.21E-01	6.06E-01	414	352215
255.2	Adrenal hypofunction	endocrine/metabolic	0.92	0.84	1.02	9.64E-02	5.21E-01	462	365405
709.3	Systemic sclerosis	Dermatologic	0.84	0.74	0.94	5.04E-03	6.81E-02	275	354765

Table S2. Associations in males between polygenic risk score of refractory celiac disease-type II and the autoimmune disease phenotypes using the PRS-PheWAS.

Phenotype	Description	Group	OR	95%LCI	95%UCI	P value	FDR	n_cases	n_controls
709.4	Polymyositis	Dermatologic	1.16	0.79	1.72	4.59E-01	8.65E-01	23	161980
715.2	Ankylosing spondylitis	musculoskeletal	1.11	1.00	1.22	2.77E-02	1.33E-01	467	164288
695.42	Systemic lupus erythematosus	Dermatologic	1.06	0.87	1.29	5.98E-01	8.74E-01	89	161723
250.1	Type 1 diabetes	endocrine/metabolic	1.04	1.00	1.08	4.72E-02	1.53E-01	2145	149602
555.1	Regional enteritis	Digestive	1.02	0.96	1.08	4.22E-01	8.65E-01	1104	134245
242.1	Graves' disease	endocrine/metabolic	1.02	0.87	1.19	8.41E-01	8.75E-01	154	163993
555	Inflammatory bowel disease and other gastroenteritis and colitis	Digestive	1.01	0.97	1.05	4.90E-01	8.65E-01	3127	134245
555.2	Ulcerative colitis	Digestive	1.01	0.97	1.05	6.72E-01	8.74E-01	2344	134245
695.4	Lupus (localized and systemic)	Dermatologic	1.00	0.84	1.19	9.98E-01	9.98E-01	116	161723
696.4	Psoriasis	Dermatologic	0.99	0.95	1.03	7.41E-01	8.75E-01	2480	160447
446	Polyarteritis nodosa and allied conditions	circulatory system	0.99	0.92	1.07	8.31E-01	8.75E-01	664	160518
335	Multiple sclerosis	Neurological	0.99	0.92	1.07	8.33E-01	8.75E-01	500	158318
939	Atopic/contact dermatitis due to other or unspecified	Dermatologic	0.99	0.95	1.03	4.99E-01	8.65E-01	2540	166463
697	Sarcoidosis	Dermatologic	0.98	0.89	1.08	6.73E-01	8.74E-01	439	162188
714.1	Rheumatoid arthritis	musculoskeletal	0.97	0.93	1.01	2.09E-01	4.94E-01	1783	164288
694.1	Vitiligo	Dermatologic	0.97	0.75	1.25	8.01E-01	8.75E-01	61	162188
695.41	Cutaneous lupus erythematosus	Dermatologic	0.94	0.72	1.24	6.66E-01	8.74E-01	50	161723
281.11	Pernicious anemia	Hematopoietic	0.93	0.85	1.03	1.61E-01	4.20E-01	436	153586
245.21	Chronic lymphocytic thyroiditis	endocrine/metabolic	0.91	0.64	1.30	6.05E-01	8.74E-01	33	163993
571.6	Primary biliary cirrhosis	Digestive	0.86	0.69	1.07	1.41E-01	4.09E-01	96	159840
255.2	Adrenal hypofunction	endocrine/metabolic	0.85	0.74	0.98	3.08E-02	1.33E-01	191	166669
709	Diffuse diseases of connective tissue	Dermatologic	0.85	0.75	0.95	1.02E-02	8.76E-02	258	161980
709.7	Unspecified diffuse connective tissue disease	Dermatologic	0.78	0.62	0.99	3.57E-02	1.33E-01	81	161980
709.2	Sicca syndrome	Dermatologic	0.75	0.61	0.94	9.06E-03	8.76E-02	96	161980
709.3	Systemic sclerosis	Dermatologic	0.66	0.48	0.90	7.79E-03	8.76E-02	48	161980
714.2	Juvenile rheumatoid arthritis	musculoskeletal	0.50	0.29	0.86	1.35E-02	8.76E-02	18	164288
709.5	Dermatomyositis	Dermatologic	Non estimable						

Table S3. Associations in females between polygenic risk score of refractory celiac disease-type II and the autoimmune disease phenotypes using the PRS-PheWAS.

Phenotype	Description	Group	OR	95%LCI	95%UCI	P value	FDR	n_cases	n_controls
714.2	Juvenile rheumatoid arthritis	musculoskeletal	1.41	1.05	1.89	1.91E-02	2.48E-01	42	194690
715.2	Ankylosing spondylitis	musculoskeletal	1.18	1.05	1.33	7.85E-03	2.04E-01	235	194690
245.21	Chronic lymphocytic thyroiditis	endocrine/metabolic	1.12	1.01	1.23	3.84E-02	3.33E-01	322	179428
555.1	Regional enteritis	Digestive	1.05	0.99	1.11	7.09E-02	3.68E-01	1345	156578
335	Multiple sclerosis	Neurological	1.04	0.98	1.10	1.47E-01	5.47E-01	1294	192193
709.7	Unspecified diffuse connective tissue disease	Dermatologic	1.04	0.91	1.19	5.71E-01	9.28E-01	221	192785
281.11	Pernicious anemia	Hematopoietic	1.02	0.96	1.08	5.15E-01	9.28E-01	889	181973
696.4	Psoriasis	Dermatologic	1.01	0.97	1.05	4.99E-01	9.28E-01	2153	190073
555	Inflammatory bowel disease and other gastroenteritis and colitis	Digestive	1.01	0.97	1.05	4.78E-01	9.28E-01	3165	156578
697	Sarcoidosis	Dermatologic	1.01	0.92	1.11	7.88E-01	9.28E-01	464	193823
709.2	Sicca syndrome	Dermatologic	1.01	0.95	1.07	8.56E-01	9.28E-01	870	192785
446	Polyarteritis nodosa and allied conditions	circulatory system	1	0.94	1.06	9.74E-01	9.74E-01	1053	196006
555.2	Ulcerative colitis	Digestive	0.99	0.95	1.03	7.76E-01	9.28E-01	2137	156578
709	Diffuse diseases of connective tissue	Dermatologic	0.99	0.93	1.05	7.67E-01	9.28E-01	1311	192785
695.41	Cutaneous lupus erythematosus	Dermatologic	0.99	0.86	1.14	9.08E-01	9.44E-01	197	192607
939	Atopic/contact dermatitis due to other or unspecified	Dermatologic	0.99	0.95	1.03	6.66E-01	9.28E-01	2643	198656
250.1	Type 1 diabetes	endocrine/metabolic	0.99	0.93	1.05	6.57E-01	9.28E-01	1574	189026
714.1	Rheumatoid arthritis	musculoskeletal	0.98	0.94	1.02	3.04E-01	8.78E-01	3820	194690
694.1	Vitiligo	Dermatologic	0.98	0.81	1.19	8.47E-01	9.28E-01	101	193823
255.2	Adrenal hypofunction	endocrine/metabolic	0.98	0.87	1.10	7.22E-01	9.28E-01	271	198736
571.6	Primary biliary cirrhosis	Digestive	0.97	0.86	1.09	5.60E-01	9.28E-01	318	192375
695.42	Systemic lupus erythematosus	Dermatologic	0.96	0.87	1.06	4.49E-01	9.28E-01	456	192607
695.4	Lupus (localized and systemic)	Dermatologic	0.95	0.88	1.03	2.61E-01	8.48E-01	552	192607
709.4	Polymyositis	Dermatologic	0.95	0.71	1.28	7.28E-01	9.28E-01	44	192785
242.1	Graves' disease	endocrine/metabolic	0.94	0.87	1.02	1.39E-01	5.47E-01	617	179428
709.3	Systemic sclerosis	Dermatologic	0.88	0.77	1.01	6.33E-02	3.68E-01	227	192785
709.5	Dermatomyositis	Dermatologic	Non estimable						

Table S4. SNP-by-SNP analysis between PRS-RCDII and ankylosing spondylitis and systemic sclerosis.

SNP	Outcome	OR	95%LCI	95%UCI	Pvalue
rs2041570	Ankylosing spondylitis	1.05	0.98	1.13	0.172
rs7324708	Ankylosing spondylitis	1.09	1.01	1.17	0.019
rs205047	Ankylosing spondylitis	1.10	1.03	1.18	0.0046
rs2041570	Systemic sclerosis	0.87	0.77	0.99	0.032
rs7324708	Systemic sclerosis	0.95	0.84	1.07	0.404
rs205047	Systemic sclerosis	0.87	0.76	0.98	0.041
